# Glutathione attenuates sepsis-associated encephalopathy via dual modulation of NF-κB and PKA/CREB pathways

**DOI:** 10.1515/med-2025-1282

**Published:** 2025-09-20

**Authors:** Cailin Wang, Yong Liu, Xiangru Wen, Hui Lu

**Affiliations:** Medical Research Center, People’s Hospital of Suzhou New District, 215000, Suzhou, Jiangsu, China; Department of Orthopedics, People’s Hospital of Suzhou New District, 215000, Suzhou, Jiangsu, China; Research Center for Neurobiology and Department of Neurobiology, Xuzhou Medical College, 215000, Xuzhou, Jiangsu, China; Department of Pain, Suzhou Hospital of Integrated Traditional Chinese and Western Medicine, 215000, Suzhou, Jiangsu, China

**Keywords:** sepsis, SAE, GSH, PKA, NF-κB

## Abstract

**Background:**

Sepsis-associated encephalopathy (SAE) remains a major unmet clinical need, due to the lack of effective treatments. Although glutathione (GSH) is known for its antioxidant properties, its specific neuroprotective role via modulation of inflammatory pathways in SAE remains poorly understood.

**Methods:**

Using a lipopolysaccharide (LPS)-induced murine sepsis model, we examined GSH’s effects through comprehensive behavioral assessments, histopathological analysis, and molecular profiling. Mice were pretreated with GSH prior to LPS challenge, and outcomes were assessed across multiple parameters.

**Key results:**

This study reveals that GSH pretreatment significantly improved survival rates by 10% (*p* < 0.01) and significantly alleviates neurological deficits in a murine model of sepsis. Behaviorally, GSH reversed depression-like symptoms, boosting locomotor activity (*p* = 0.009) and reducing immobility (*p* < 0.05). Histopathological analysis showed that GSH preserved hippocampal structure, maintaining 40% more viable neurons in CA1/DG regions (*p* < 0.05). Mechanistically, GSH exerts dual neuroprotective actions: it robustly suppresses NF-κB signaling via inhibition of p65 nuclear translocation and downregulation of IL-1β and IL-6, while simultaneously activating the PKA/CREB pathway revealing a previously unrecognized mechanism of action.

**Conclusion:**

This study provides the first evidence of GSH’s dual mechanism action in SAE, establishing it as a promising multi-target therapeutic candidate. These findings open new avenues for developing effective SAE interventions targeting both inflammatory and neuroprotective pathways.

## 1 Introduction

Sepsis-associated encephalopathy (SAE) is a critical complication affecting the central nervous system in patients with sepsis, characterized by diffuse or multifocal brain dysfunction secondary to systemic infection [1]. Notably, SAE can develop even in the absence of direct brain infection, rendering its pathophysiology particularly complex [2]. SAE is common among critically ill patients in the intensive care unit (ICU), affecting up to 70% of individuals with severe systemic infections [3]. Its clinical manifestations range from mild disturbances, such as confusion and attention deficits, to profound coma, with patients often experiencing behavioral, cognitive, arousal, and consciousness impairments [4]. Furthermore, many patients develop long-term cognitive deficits [5]. Despite its high prevalence in ICU settings, the pathogenesis of SAE remains poorly understood, and there are no effective treatments currently available [6]. Thus, SAE represents a significant clinical challenge, drawing increasing attention from the global medical community.

In addition to cognitive dysfunction, depression is one of the most debilitating complications of sepsis-induced brain dysfunction, contributing to increased morbidity and mortality [7]. Depression in this context remains a major therapeutic challenge, as current treatments primarily alleviate symptoms without addressing the underlying causes. This underscores the urgent need to elucidate the mechanisms underlying sepsis-induced depression and to identify novel therapeutic strategies targeting these behavioral impairments [8].

Inflammatory mediators – including cytokines, the complement system, and disrupted intracellular signaling pathways – are believed to play key roles in the pathogenesis of SAE. Neuroinflammation, characterized by activation of microglia, astrocytes, and neurons, leads to a dysregulated balance between pro- and anti-inflammatory responses [9]. Moreover, activation of the NF-κB signaling pathway and excessive generation of reactive oxygen species (ROS) have been implicated in SAE development [10]. Glutathione (GSH), a crucial endogenous antioxidant, is essential for counteracting oxidative stress during sepsis [11].

GSH, a naturally occurring tripeptide, contributes to immune regulation, redox homeostasis, apoptosis, and modulation of intracellular signaling cascades [12]. Previous studies suggest that GSH improves outcomes in sepsis, particularly in organ systems such as the lungs, liver, and kidneys [13]. However, its specific role in protecting the brain during sepsis – particularly regarding neuroinflammation and neuronal damage – remains underexplored.

Taken together, we hypothesize that GSH may attenuate neuroinflammation and hippocampal neuronal injury by enhancing the cAMP/PKA signaling pathway and inhibiting NF-κB activation, thereby alleviating depressive-like behavior associated with SAE. This study aims to investigate the therapeutic potential of GSH in sepsis-induced brain injury, offering novel insights into its application as a candidate treatment for SAE.

## 2 Methods

### 2.1 Reagents

Lipopolysaccharide (LPS) and GSH were purchased from Sigma-Aldrich (St Louis, MO, USA). The following primary antibodies were used: anti-PKA, polyclonal anti-p-PKA (Thr198), anti-IL-1β, anti-IL-6, anti-NF-κB, mouse monoclonal anti-Caspase-3 (CASP3), and goat monoclonal anti-IL-10, all obtained from Santa Cruz Biotechnology (Santa Cruz, CA, USA). A rabbit polyclonal anti-cleaved-Caspase-3 (Cleaved-CASP3) antibody was purchased from Cell Signaling Technology (Beverly, MA, USA). The secondary antibodies used in this study were also purchased from Sigma-Aldrich.

### 2.2 Animal model

Male C57BL/6 mice (2–3 months, 20 ± 5 g) were obtained from the Experimental Animal Center of Xuzhou Medical College. They were housed under standard conditions with free access to food and water at 21°C on a 12 h light/dark cycle. All procedures were approved by the Institutional Animal Care and Use Committee (IACUC) of Xuzhou Medical University. Sepsis model: sepsis was induced by a single intraperitoneal (i.p.) injection of LPS (25 mg/kg) dissolved in normal saline. Mice in the control (CON) group received an equivalent volume of saline. GSH treatment: mice in the GSH treatment group received daily i.p. injections of GSH (100 mg/kg) for 4 days before LPS administration (details in Figure S1).

### 2.3 Pathological behavioral scoring

Pathological behavioral scoring was conducted to assess the effects of LPS injection on mice, focusing on piloerection, ptosis, and activity level. Piloerection was scored as 0 (no piloerection), 1 (moderate piloerection), or 2 (extensive piloerection). Ptosis was scored as 0 (normal or >50% open), 1 (slight ptosis, 1/3–1/2 closed), or 2 (severe ptosis, <1/3 open). Activity was scored as 0 (active behavior), 1 (inactivity), or 2 (lethargy or curled up). This scoring was used to evaluate the impact of LPS and treatments, minimizing external disturbances during assessment (Table S1).

### 2.4 Open-field (OFT) and closed-field test (CFT)

Depression-like behavior was assessed using the OFT and CFT based on the method of Prut and Belzung. OFT: mice were placed at the center of an open-field apparatus (50 × 50 × 30 cm) and allowed to acclimate for 3 min. Their free-moving behavior was then recorded for 5 min using an open-field tracking system. Activity was quantified in terms of total movement time, total distance traveled, distance in the central area, and time spent in the central area. CFT: following the OFT, mice were placed in a dark, closed-box of the same size, and behavior was recorded as in the OFT. The experiment was conducted in a quiet environment to minimize disturbances [14].

### 2.5 Elevated plus maze (EPM) test

The EPM, used to assess depression-like behavior, consists of a cross-shaped elevated platform (64 × 64 cm) with two open arms and two closed arms. Mice were acclimated to the test room for 1 h before being placed at the center of the maze, facing the open arm. They were allowed to explore for 5 min while their behavior was recorded. The system tracked entries into the open and closed arms and time spent in the open arms. Parameters calculated included the percentage of time spent in the open arms and total entries into both arms. After each trial, the maze was cleaned with 75% ethanol and allowed to dry before the next test. The experiment was conducted in a quiet environment to minimize disturbances [15].

### 2.6 Tail suspension test

In the tail suspension test, the tip of the mouse’s tail (1.0–1.5 cm) was affixed to a metallic surface using medical adhesive tape, and the mouse was suspended 30 cm above the surface for 6 min under 100 lux illumination. During the test, two parameters were recorded: immobility, defined as the total time the mouse remained immobile for more than 2 s, and latency, the time taken for the mouse to first exhibit immobility after being suspended [16].

### 2.7 Nissl staining and HE staining

Mice were transcardially perfused with 200 mL ice-cold phosphate-buffered saline (PBS), followed by 300 mL of 10% formalin. After 48 h of fixation, brains were dehydrated, embedded in paraffin, and sectioned into 10 µm slices. Hippocampal sections were stained with Nissl stain (Solarbio, China) following the manufacturer’s instructions. Surviving pyramidal neurons in the CA1–CA3 and dentate gyrus (DG) regions were counted at ×400 magnification. For HE staining, 5 µm brain sections were prepared to assess hippocampal cell damage [17].

### 2.8 Immunohistochemistry

Frozen mouse brain sections (30 μm) were washed 3–4 times with 0.1 M PBS for 5 min each, and then incubated in blocking solution containing 5% bovine serum albumin for 2 h at room temperature. After blocking, sections were incubated overnight at 4°C with primary antibodies: anti-GFAP (1:100; Abcam) and either anti-NeuN or anti-BrdU (1:150) in 10% goat serum diluted in PBS. The next day, sections were washed 3–4 times with PBS for 5 min each and incubated with appropriate anti-rabbit secondary antibodies for 2 h at room temperature. After washing, the sections were counterstained with DAPI (1:2,000; Sigma) for 3 min to label the nuclei. Finally, sections were mounted and observed under a fluorescence microscope.

### 2.9 Western blot analysis

Hippocampal tissues from each group of ten mice were homogenized in RIPA lysis buffer containing 1% PMSF. After adding SDS loading buffer, samples were boiled for 5 min and proteins were separated by SDS-PAGE. Following electrophoresis, proteins were transferred to a membrane, which was blocked with skimmed milk to reduce nonspecific binding. The membrane was incubated overnight at 4°C with primary antibodies (PI3K, P-PI3K, Pan-AKT, AKT1, caspase-3, and cleaved caspase-3). After washing with buffer, the membrane was incubated with a secondary antibody (1:200) for 2 h. Protein bands were scanned and analyzed using Quantity One software (BIO-RAD, USA) [18].

### 2.10 Statistical analysis

Densitometric analysis of immunofluorescence and western blot bands was performed using ImageJ software (National Institutes of Health, Bethesda, MD, USA). Data were analyzed with GraphPad Prism 7 (GraphPad Software, CA, USA) and images were processed using Photoshop CS6. Statistical analyses were conducted using SPSS version 13.0 for Windows. Data are presented as mean ± SEM. One-way ANOVA followed by the Newman–Keuls test was used for statistical comparisons, with *P* < 0.05 considered significant. In behavioral tests, all animals were treated as independent samples.


**Ethical approval:** All animal experiments were conducted in accordance with the National Institutes of Health Guide for the Care and Use of Laboratory Animals (8th edition, 2011) and approved by the Institutional Animal Care and Use Committee (IACUC) of People’s Hospital of Suzhou New District (Approval No. L2022603).

## 3 Result

### 3.1 GSH significantly improves mortality and pathological behavior in LPS-induced sepsis model

**Figure 1:**
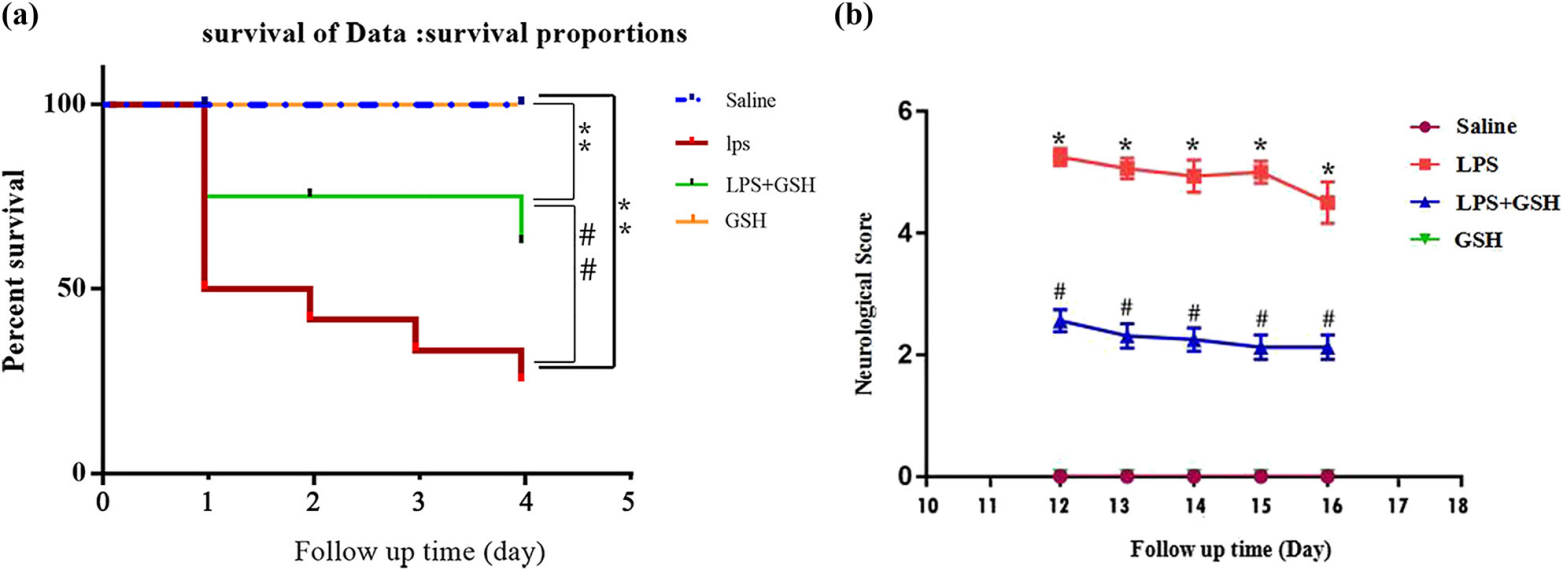
Survival curve and neurological scores (a) survival curve showing the survival rates of mice from four groups over 4 days post-LPS injection: Saline (blue), LPS (red), LPS + GSH (green), and GSH (yellow). (b) Neurological scores over time for the four groups: saline (●, purple), LPS (■, red), LPS + GSH (▲, blue), and GSH (▼, green). Data are presented as mean ± SEM (*n* = 12). *P < 0.05 vs saline, ^#^ *P* < 0.05 vs LPS.

To assess the effect of GSH pretreatment on sepsis-associated mortality, mortality within 6 days of LPS injection were recorded (Figure 1a). Survival analysis showed that 24 h after LPS injection, the survival rate in the LPS group was 50%, whereas the LPS + GSH group exhibited a higher survival rate of 60%. Significant differences were observed between the groups (NS vs LPS: *p* < 0.01; LPS vs LPS + GSH: *p* < 0.01). Pathological scoring on Day 12 revealed functional impairments in both LPS and LPS + GSH groups, but no deficits in saline and GSH groups. The LPS group exhibited significantly higher pathological scores than the saline group (*p* < 0.001), and the scores were also significantly higher than those in the LPS + GSH group (*p* < 0.001), indicating a protective effect of GSH (Figure 1b).

### 3.2 Effect of GSH on depression-like behavior of LPS-induced SAE model

**Figure 2:**
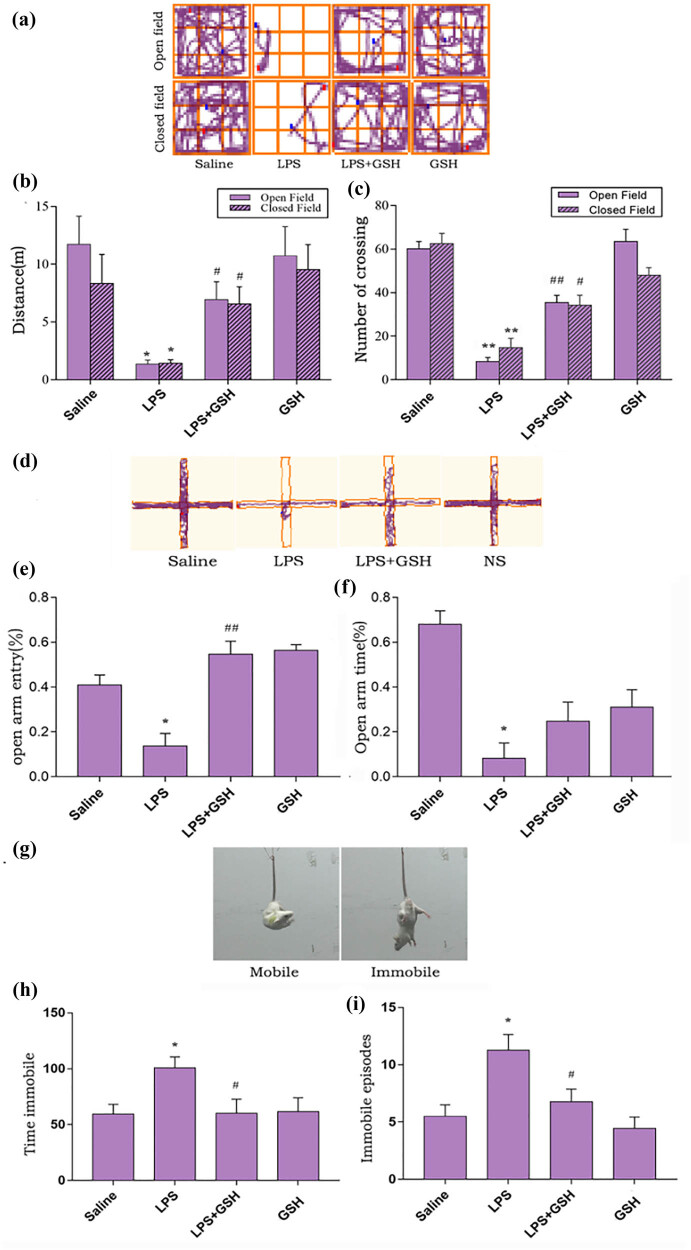
Behavioral test. (a) The autonomous trajectory map of mice in the open and closed fields after 14 days of LPS (i.p.) injection. (b) Total distance traveled by mice in open and closed fields. (c) Number of crossings in the open and closed fields. (d) Autonomous trajectory map of mice in the cross maze 24 h after LPS (i.p.) injection. (e) Percentage of time spent in the open arm. (f) Percentage of time spent in the open arm during the median time. (g) The mobile and immobile behaviors of mice during suspension tests. (h) Time spent still. (i) Number of times the mouse remained still. Data are expressed as mean ± SEM (*n* = 20). *P < 0.05 vs saline group, ^#^ *P* < 0.05 vs LPS group.

To assess the protective effect of GSH against LPS-induced depression-like behavior, a battery of behavioral tests was performed, including the OFT, CFT, elevated plus maze, and tail suspension test. In the OFT and CFT, LPS-treated mice exhibited reduced locomotion and fewer crossings compared to controls (total distance: *P* = 0.008; number of crossings: *P* < 0.001). GSH pretreatment significantly restored spontaneous activity, as evidenced by increased total distance traveled in the open field (*P* = 0.009) and more crossings compared to the LPS group (*P* < 0.001) (Figure 2a–c). In the elevated plus maze, LPS-treated mice spent less time in the open arms (*P* = 0.001) and made fewer entries (*P* = 0.046), indicative of anxiety-like behavior. GSH treatment increased both the time spent (*P* = 0.143) and the number of entries (*P* < 0.001) into the open arms (Figure 2d–f). The tail suspension test further confirmed GSH’s impact. LPS-treated mice showed increased immobility (*P* < 0.05), a hallmark of depression-like behavior, which was significantly reduced following GSH treatment (*P* < 0.05). These findings demonstrate that GSH effectively alleviates depression-like and sickness behaviors in the LPS-induced sepsis model (Figure 2g–i).

### 3.3 GSH improves the damage of neurons

**Figure 3:**
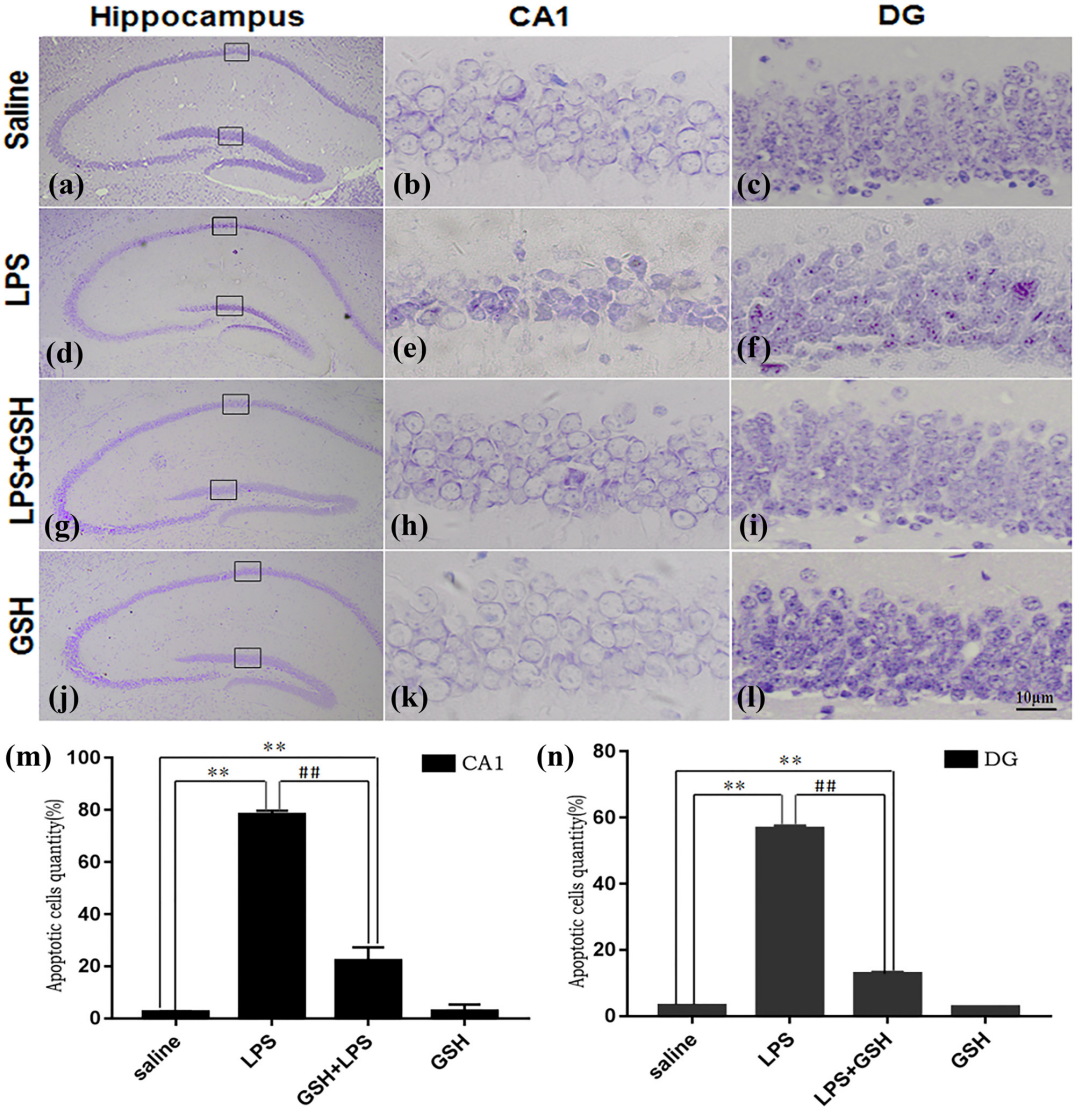
Nissl staining of hippocampal CA1 and DG regions. (a)–(c) Saline group: sections of the hippocampal CA1 and DG regions. (d)–(f) LPS group: Sections of the hippocampal CA1 and DG regions. (g)–(i) LPS + GSH group: Sections of the hippocampal CA1 and DG regions. (j)–(l) GSH group: Sections of the hippocampal CA1 and DG regions. (m) Quantification of apoptotic cells in the CA1 region. (n) Quantification of apoptotic cells in the DG region. Images were acquired at ×40 magnification. Data are expressed as mean ± SEM (*n* = 6). *P < 0.05 vs saline group, ^#^ *P* < 0.05 vs LPS group.

**Figure 4:**
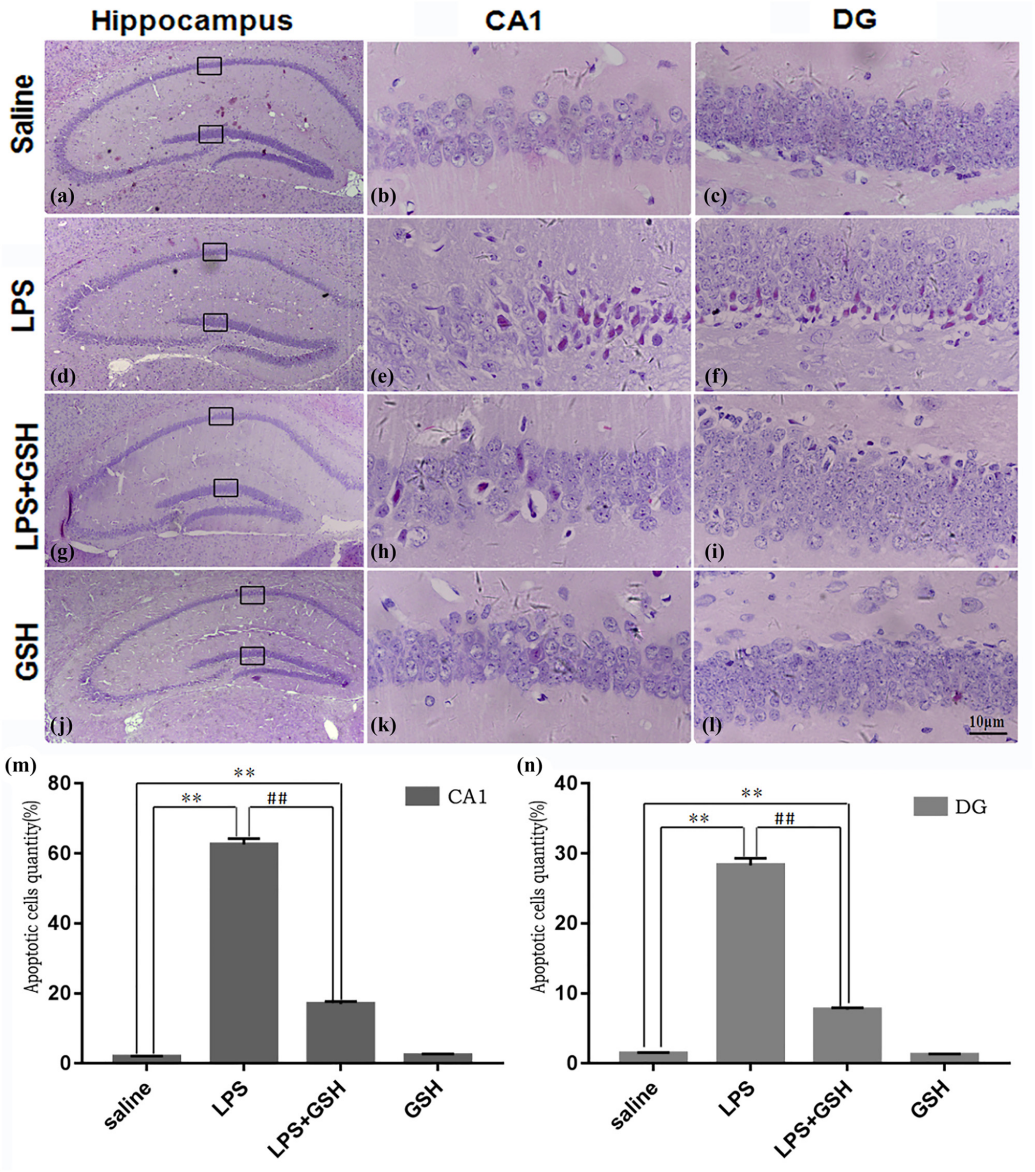
HE staining of hippocampal CA1 and DG regions. (a)–(c) Saline group: Sections of the hippocampal CA1 and DG regions. (d)–(f) LPS group: Sections of the hippocampal CA1 and DG regions. (g)–(i) LPS + GSH group: Sections of the hippocampal CA1 and DG regions. (j)–(l) GSH group: Sections of the hippocampal CA1 and DG regions. (m) Quantification of apoptotic cells in the CA1 region. (n) Quantification of apoptotic cells in the DG region. Images were acquired at ×40 magnification. Data are expressed as mean ± SEM (*n* = 6). *P < 0.05 vs saline group, ^#^ *P* < 0.05 vs LPS group.

**Figure 5:**
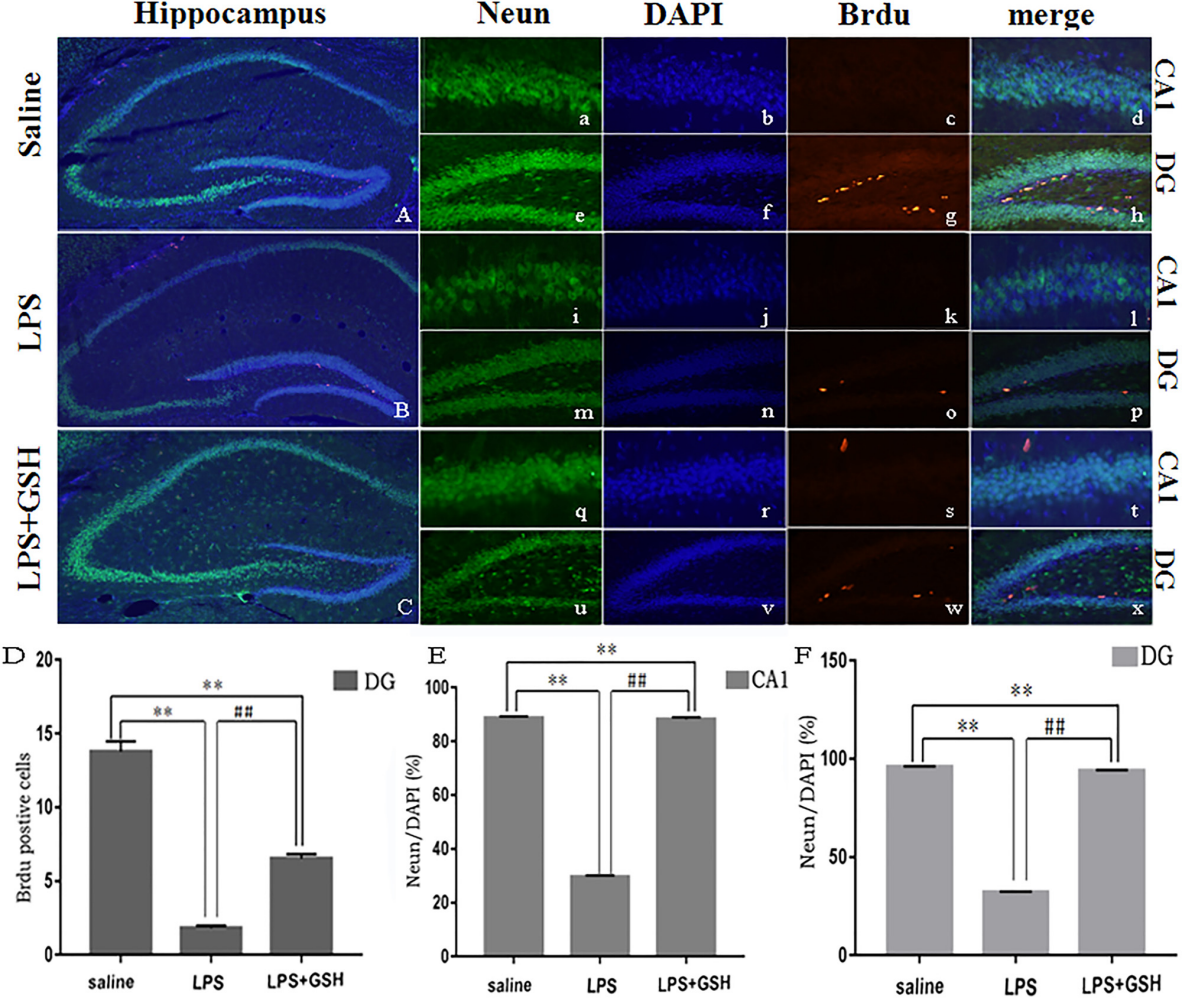
Immunofluorescence staining. (A) Representative photomicrographs of the saline group. Panels (a–d) show NEUN, BRDU, DAPI, and merged staining of the hippocampal CA1 region (×40). Panels (e–h) show fluorescent staining of NEUN, BRDU, DAPI, and merge of the hippocampal DG region. (B) Representative photomicrographs of the LPS group. Panels (i–l) show NEUN, BRDU, DAPI, and merged staining of the hippocampal CA1 region (×40). Panels (m–p) show fluorescent staining of NEUN, BRDU, DAPI, and merge of the hippocampal DG region. (C) Representative photomicrographs of the LPS + GSH group. Panels (q–t) show NEUN, BRDU, DAPI, and merged staining of the hippocampal CA1 region (×40). Panels (u–x) show fluorescent staining of NEUN, BRDU, DAPI, and merge of the hippocampal DG region. (D) Quantitative analysis of the number of BrdU-positive cells per unit area in the hippocampal DG. (E) Quantitative analysis of NEUN and DAPI ratio per unit area of the hippocampal CA1. (F) Quantitative analysis of NEUN and DAPI ratio per unit area of the hippocampal DG. Data are expressed as mean ± SEM (*n* = 6). *P < 0.05 vs saline group, ^#^ *P* < 0.05 vs LPS group.

GSH treatment markedly attenuated hippocampus neuronal damage in the LPS-induced sepsis model. Nissl and HE staining revealed pronounced neuronal degeneration in the CA1 region following LPS injection, characterized by disorganized neurons, blurred Nissl bodies, and irregular cellular structures. However, GSH pretreatment alleviated neuronal degeneration, preserved neuron morphology, and enhanced cell survival. Compared to the LPS group, the GSH-treated group exhibited fewer signs of cellular damage, with more intact pyramidal neurons and less nuclear shrinkage (Figures 3 and 4). Immunofluorescence staining for NeuN further confirmed these results, showing a notable increase in the number of surviving neurons in the GSH-treated group compared to the LPS group. Although neuronal survival in the GSH group did not reach control levels, it was significantly higher than that in the LPS group, underscoring the neuroprotective effect of GSH against LPS-induced hippocampal damage (Figure 5).

### 3.4 Protective effects of GSH against LPS-induced neuronal damage and inflammation in sepsis

**Figure 6:**
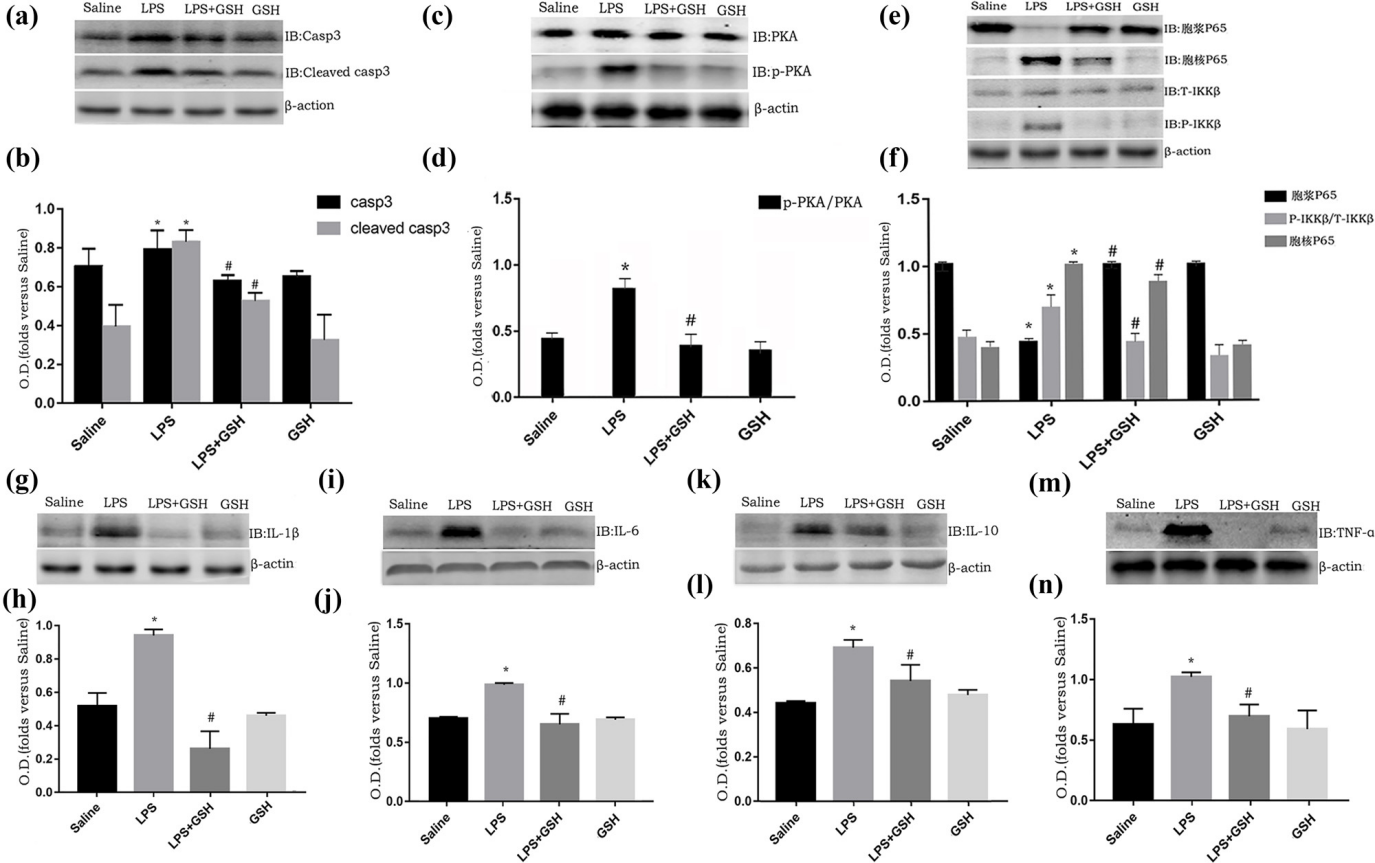
Western blot. (a) and (b) Activation of Caspase 3 and Cleaved Caspase 3 in the hippocampus 14 days after LPS injection was analyzed by immunoblot. (a) Scanning immunoblot bands. (b) The intensity of the bands is expressed as optical density (OD). (c) and (d) Expression changes in P65 in the cytoplasm and phosphorylation levels of IKKβ 14 days after LPS injection, analyzed by immunoblot. (c) Scanning immunoblot bands. (d) The intensity of the bands is expressed as OD. (e) and (f) Immunoblot analysis of PKA and p-PKA levels in the hippocampus 14 days after LPS injection. (e) Scanning immunoblot bands. (f) The intensity of the bands is expressed as O.D. (g)–(n) Expression of IL-1β, IL-6, IL-10, and TNF-α in the hippocampus 4 days after LPS injection was analyzed by immunoblot. (d) Scanning immunoblot bands. (d) The intensity of the bands is expressed as OD. (Data are expressed as mean ± SEM, *n* = 6. *P < 0.05 vs saline group, ^#^ *P* < 0.05 vs LPS group).

To investigate the protective effects of GSH on the LPS-induced sepsis model, we focused on its impact on neuronal damage, inflammation, and key cellular signaling pathways. Our findings demonstrated that GSH significantly reduced neuronal apoptosis in the hippocampus. This was evidenced by decreased levels of caspase-3 and cleaved caspase-3 in the LPS + GSH group compared to the LPS group (*P* < 0.05), suggesting that GSH contributes to neuronal preservation in the context of sepsis (Figure 6a and b).

Beyond its anti-apoptotic effects, GSH also attenuated the inflammatory response. GSH treatment significantly reduced the hippocampal expression of pro-inflammatory cytokines, including TNF-α (Figure 6m and n), IL-1β (Figure 6g and h), and IL-6 (Figure 6i and j, *P* < 0.05), further supporting its anti-inflammatory effects. These effects are likely mediated by inhibition of the NF-κB pathway. GSH pretreatment lowered the phosphorylation of IKKβ in the cytoplasm and prevented the nuclear translocation of p65, indicating suppression of NF-κB activation (Figure 6e and f, *P* < 0.05).

Moreover, GSH activated the PKA signaling pathway, which is implicated in regulating cellular responses to inflammation. Phosphorylation levels of PKA and CREB were significantly higher in the LPS + GSH group compared to the LPS group (*P* < 0.05), suggesting that GSH may also exert its protective effects through the activation of this pathway (Figure 6c and d).

Overall, GSH protects against LPS-induced neuronal damage and inflammation, likely through the modulation of multiple signaling pathways, including NF-κB and PKA. These findings highlight GSH’s potential as a therapeutic agent in sepsis-related brain injury.

## 4 Discussion

SAE is a frequent and severe complication of sepsis, often leading to cognitive decline and affective disorders in survivors. Despite its clinical relevance, the pathophysiological mechanisms underlying SAE remain incompletely understood, and effective targeted therapies are lacking [19]. In this study, we investigated the neuroprotective potential of GSH in a murine model of LPS-induced sepsis and our findings reveal that GSH exerts both anti-inflammatory and neuroprotective effects in the context of SAE.

We first demonstrated that prophylactic administration of GSH significantly improved the survival rate of septic mice, with a 10% increase compared to the untreated LPS group. This enhancement in survival suggests that GSH mitigates systemic inflammation and potentially reduces sepsis-induced multi-organ dysfunction [20]. In addition to improving survival, GSH treatment mitigated behavioral abnormalities and reduced neurological deficits in septic mice. These observations support the hypothesis that GSH confers protection against sepsis-induced brain injury, especially those associated with hippocampal damage – a region critically involved in memory and emotional regulation [21].

Behavioral assessments revealed that GSH significantly alleviated depression-like symptoms in septic mice. Compared to the LPS group, GSH-treated mice exhibited increased locomotor activity and reduced immobility in both OFT and CFTs, indicating improved motivation and exploratory behavior. These behavioral improvements were accompanied by histological evidence of preserved neuronal structure in the hippocampal CA1 and DG regions. These findings strongly suggest that GSH supports hippocampal structural integrity and confers resilience to SAE-related neurodegeneration [22].

At the molecular level, we observed that GSH significantly reduced the hippocampal expression of key pro-inflammatory cytokines, including TNF-α, IL-1β, and IL-6. These cytokines are key mediators of neuroinflammation and have been strongly implicated in SAE pathogenesis [23]. By downregulating these inflammatory mediators, GSH may directly counteract the neurotoxic inflammatory cascade triggered by systemic sepsis. Moreover, our results suggest that the anti-inflammatory effects of GSH are, at least in part, mediated by inhibition of the NF-κB signaling pathway. NF-κB is a well-established transcriptional regulator of inflammatory gene expression. We found that GSH suppressed the phosphorylation and nuclear translocation of the p65 subunit of NF-κB, thereby reducing the transcriptional activation of pro-inflammatory genes [24]. This mechanism likely contributes to the observed reduction in cytokine levels and neuronal injury in GSH-treated mice.

In addition to its anti-inflammatory effects, GSH is also recognized as a key intracellular antioxidant. Although we did not directly measure oxidative stress markers – such as ROS, lipid peroxidation, or the GSH/GSSG ratio – it is reasonable to consider that its antioxidant properties may have played a role in the neuroprotection observed in this study. Previous reports have highlighted the importance of GSH in limiting oxidative damage in neuroinflammatory conditions, including LPS-induced brain injury [25,26]. Future work involving the assessment of oxidative stress parameters will be important to clarify how much of GSH’s protective effect is driven by redox regulation versus inflammation control.

We also found that GSH treatment led to increased phosphorylation of CREB in the hippocampus, suggesting activation of the PKA/CREB signaling pathway, which is known to support neuronal survival and plasticity. However, we were not able to examine downstream CREB-regulated genes such as BDNF or c-fos in the current study. Given the well-established role of BDNF in maintaining cognitive function and synaptic health  [27], it is possible that this pathway contributed to the observed benefits. Further studies using gene expression or protein analysis will be needed to explore this mechanism in more detail.

One important limitation of this study is the use of a prophylactic treatment strategy, where GSH was administered before sepsis induction. While this approach allowed us to assess its potential protective effects under controlled experimental conditions, it does not fully reflect clinical situations in which treatment typically begins after the onset of infection. This limits the direct translational relevance of our findings. Nonetheless, prophylactic models remain a common and useful tool in early-stage research for identifying promising therapeutic targets. Future studies should evaluate the effectiveness of GSH when given after sepsis onset to better understand its potential for clinical use.

## 5 Conclusions

In summary, this study demonstrates that GSH pre-treatment offers significant neuroprotection in a murine model of sepsis. By attenuating neuroinflammation and preserving hippocampal neurons, GSH improves behavioral outcomes and survival in septic mice. These findings highlight the therapeutic potential of GSH for the prevention or treatment of SAE. Future studies are needed to explore the translational applicability of these findings in clinical settings and to further elucidate the molecular pathways through which GSH exerts its protective effects. Given its dual antioxidant and anti-inflammatory properties, GSH represents a promising candidate for therapeutic intervention in sepsis-associated neurological dysfunction.

## Supplementary Material

Supplementary material
