# Supplementary material for: Glutathione attenuates sepsis-associated encephalopathy via dual modulation of NF-κB and PKA/CREB pathways
Source: Open Med (Wars). 2025 Sep 20;20(1):20251282. doi: 10.1515/med-2025-1282 (PMC12452075; doi:10.1515/med-2025-1282)
Supplement: Supplementary material [file med-2025-1282-sm.pdf]

Supplementary material

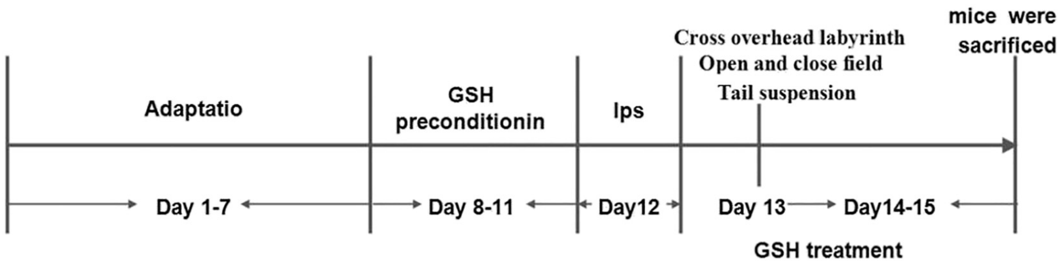

Figure S1: Experimental flowchart.

Table S1: Pathological scoring

Score	Fur standing up	Ptosis (drooping of the upper eyelid)	Activity level
0	Only appears in the waist and hip area	Eyes open more than half	Active behavior
1	Surrounding the body	Slightly drooping	Inactive
2	A lot of fur standing up	Eyes open less than one-third	Curls up and is lethargic
